# Primary Clear Cell Carcinoma of the Urinary Bladder

**DOI:** 10.1155/2014/593826

**Published:** 2014-07-02

**Authors:** Anthony Kodzo-Grey Venyo

**Affiliations:** Department of Urology, North Manchester General Hospital, Delaunays Road, Manchester, UK

## Abstract

Primary clear cell carcinoma of the urinary bladder (PCCUB) is rare. Literature review has revealed 47 cases of PCCUB which commonly affects women. The histogenesis of PCCUB is not certain and Müllerian origin and urotheilal origin have been postulated. The microscopic characteristics of PCCUB include cells with abundant clear cytoplasm, arranged in a solid, glandular, tubulocystic, or papillary pattern. The cells may be flat or cuboidal with abundant clear eosinophilic cytoplasm. Hobnailing may be evident. PCCUB, on immunohistochemistry, stain positively with pan-cytokeratin, cytokeratin 7, and CA 125. PCCUB may manifest with visible haematuria, lower urinary tract symptoms, and discharge. There is no consensus opinion regarding the best treatment option for PCCUBs and patient outcomes are not very clear. Surgery has been the adopted treatment of choice. Differential diagnoses of PCCUB include nephrogenic metaplasia, urothelial carcinoma with clear cell cytoplasm, diffuse large B-cell lymphoma, and metastatic clear cell carcinoma with the primary originating elsewhere. *Conclusions.* A thorough radiological imaging assessment is required in cases of PCCUB to exclude a primary tumour elsewhere. Urologists and oncologists should report cases of PCCUB they encounter and should enter them into a multicentric trial to ascertain the best management option.

## 1. Introduction

Clear cell carcinomas of the urinary bladder are uncommon; hence most clinicians would be unfamiliar with their clinical features and diagnostic characteristics which are summarized in the overview which constitutes the first part of the paper. Miscellaneous narrations from a number of reported cases of primary clear cell carcinoma of the urinary bladder have been summarized in the second part of the paper in order to vividly elucidate further details of the biological behavior of the tumour.

## 2. Overview

### 2.1. Definition

Clear cell carcinoma of the urinary bladder has been defined as a morphological variant of adenocarcinoma of the urinary bladder which mimics the Müllerian type of clear cell carcinoma of the female genital tract [1].

### 2.2. Other Terminologies

Clear cell carcinoma of the urinary bladder has also been referred to as (a) mesonephric or mesonephroid carcinoma or (b) adenocarcinoma of the urinary bladder [1].

### 2.3. Aetiology

Postulates that have been promulgated regarding the aetiology of clear cell carcinoma of the urinary bladder include the following.

- It emanates from Müllerian elements in the urinary bladder (endometriosis).
- It represents a peculiar variant of vesical adenocarcinoma of non-Müllerian derivation.
- It represents a peculiar morphologic expression of urothelial carcinoma, with glandular differentiation often uncertain [2].

### 2.4. Incidence

Few cases of clear cell carcinoma of the urinary bladder have been reported in patients whose ages have ranged between 19 years and 80 years and they have been more commonly reported in females than in males [3] (Lu et al. [3]; also see Table 1).

### 2.5. Clinical Peculiarities

Clear cell carcinomas of the urinary bladder

- tend to be high staged tumours of the urinary bladder and the stage of the tumour is an important prognostic factor [1];
- they usually occur in the urinary bladder or urethra of women [1].

### 2.6. Presentations

The reported presentations of clear cell carcinoma of the urinary bladder include (a) haematuria [4] and (b) lower urinary tract symptoms [3].

### 2.7. Macroscopic Features

Clear cell carcinomas of the urinary bladder tend to be seen as papillary and sessile lesions [1]. They present as polypoidal growths and frequently ulcerations of the urinary bladder are seen [1].

### 2.8. Microdescription (See Figures 1, 2, 3, 4, 5, and 6)

A number of features on microscopic examination of clear cell carcinoma of the urinary bladder on histological examination can be summarized as follows.

- They are often papillary or tubulocystic lesions [5].
- They are seen as flat or cuboidal neoplastic cells which have abundant clear or eosinophilic cytoplasm with glycogen and frequent hobnailing [1].
- They exhibit more pleomorphic cells and more mitotic figures than adenomatoid tumour and variable necrosis exists in them [1].
- They resemble urothelial carcinomas more than adenocarcinoma [1].
- The papillae of clear cell carcinomas of the urinary bladder at times have hyalinized cores [1].
- Myxoid stroma may be seen in the tumours [1].

### 2.9. Immunohistochemical Staining Characteristics

#### 2.9.1. Positive Stains

Clear cell carcinomas of the urinary bladder tend to exhibit positive immunohistochemical staining with the following markers [1]:

- CA125 (strong),
- CK7 (usually strong),
- Ki-67,
- p53,
- CEA,
- PAX2,
- PAX8,
- racemase (AMACR).

#### 2.9.2. Negative Stains

Clear cell carcinomas of the urinary bladder on immunohistochemical staining stain negatively with the following markers: [1, 6]

- CK20 (occasional positivity is noted),
- PSA,
- ER,
- PR [6].

### 2.10. Differential Diagnosis

Differential diagnoses of clear cell carcinoma of the urinary bladder include the following: [1]

- nephrogenic metaplasia which occurs in young age, and there is usually a history of genitourinary trauma; histological examination usually reveals minimal atypia or pleomorphism, no/rare mitotic figures, no necrosis, and no evidence of infiltrative growth [1];
- extension or metastasis from gynecological or other clear cell carcinomas [7, 8];
- urothelial carcinoma with clear cytoplasm [1];
- diffuse large B cell lymphoma [9].

With regard to differential diagnosis the characteristics of the various lesions which should be used to differentiate the various lesions from clear cell carcinoma include the following.

*Nephrogenic Adenoma*

- Gross appearance: papillary, polypoidal, and sessile structure;
- microscopic characteristics; pattern, small tubules and cysts which resemble renal tubules; hobnail cells are present; mitosis is absent; cytoplasm is scant and eosinophilic; the nuclei are bland;
- immunohistochemical staining characteristics:
   EMA (+),
   LMWCK (+),
   CK7 (+),
   CK20 (±),
   CEA (−),
   Vimentin (−),
   CA 125 (±),
   AMACR (+).

*Urothelial Carcinoma and at Times with Clear Cytoplasm* [1]

- Gross appearance: papillary structure;
- microscopic characteristics; papillae with thin cores; hobnail cells are absent; mitosis is variable; cytoplasm is moderate; nuclei exhibit mild atypia;
- immunohistochemical staining characteristics:
   EMA (+),
   LMWCK (+),
   CK7 (+),
   CK20 (+),
   CEA (+),
   Vimentin (−),
   CA125 (±).

*Metastases from Renal Cell Carcinoma*

- Gross appearance, solid polypoid growth;
- microscopic characteristics: nests with delicate vascular cores; hobnail cells absent; low mitosis; abundant and clear cytoplasm; bland nuclei;
- immunohistochemical staining characteristics:
   EMA (+),
   LMWCK (+),
   CK7 (−),
   CK20 (−),
   CEA (−),
   Vimentin (+),
   CA125 (−).

*Extension or Metastasis from Other Gynaecological Clear Cell Carcinomas [7, 8].*

*Diffuse Large B-Cell Lymphoma [9].*

### 2.11. Treatment and Prognosis

#### 2.11.1. Treatment

There is no consensus opinion regarding the best treatment option for clear cell carcinoma of the urinary bladder.

Various options of treatment that have been used for clear cell carcinoma of the urinary bladder include (see Table 1)

- transurethral resection of the lesion,
- radiotherapy,
- chemotherapy,
- partial cystectomy/diverticulectomy,
- radical surgery/total cystectomy.

It has been stated that

- the recommended treatment for primary clear cell carcinoma of the urinary bladder is surgical resection, and cystectomy is the most common procedure [3, 10, 11];
- adjuvant radiotherapy to a total dose of 50 to 60 Gy has been used as well as combination chemotherapy inclusive of cisplatin plus etoposide, doxorubicin, and cyclophosphamide [10];
- the impact of these therapeutic modalities on the long-term survival of the patient is uncertain [12–14].

#### 2.11.2. Prognosis

The natural history of clear cell carcinoma of the urinary bladder is not well understood as clear cell carcinoma of the urinary bladder is rare and the follow-up periods pursuant to treatment of reported cases have been relatively short (<5 years) [10].

Few cases of clear cell carcinoma of the urinary bladder have been reported and these have been treated with various treatment options with patients alive with no evidence of disease; some remained alive with persistent/progressive disease and others had died with disease.

Despite the fact that the prognosis of patients with clear cell carcinoma of the urinary bladder remains unclear, the survival period of the few reported cases would indicate that clear cell carcinoma of the urinary bladder may have a worse outcome than conventional urothelial carcinoma [10].

In view of the small number of tumours reported the outcome cannot at the moment be clearly documented with regard to tumour stage, grade, and option of treatment adopted but perhaps radical cystectomy plus radiotherapy and chemotherapy may improve survival; whether or not this treatment option is overtreatment can only be ascertained pursuant to a multicentric trial in the future after a large number of cases have been reported.

## 3. Discussion and Miscellaneous Narrations from Reported Cases 

Primary clear cell carcinoma of the urinary bladder was first reported by Dow and Young Jr [15] in 1968; since then, to the knowledge of the author a total of less than 50 cases of primary clear cell carcinoma of the urinary bladder have been reported (see Table 1 example of reported cases). Experience gained and lessons learnt from management of these cases have been outlined in the ensuing miscellaneous narrations. 

Sung et al. [6] integrated molecular genetic evaluation by means of fluorescent* in situ* hybridization (FISH) and X-chromosome inactivation with conventional morphologic and immunohistochemical analysis in 12 patients who had clear cell adenocarcinomas in their urinary tract. Sung et al. [6] reported the following.

- Concurrent urothelial carcinoma* in situ* was present in six cases (50%) and foci of cystitis glandularis were found in four cases (33%).
- Intestinal metaplasia and Müllerian component were not identified in any case.
- Cytoplasmic expression of *α*-methylacyl-CoA racemase was demonstrable in 10 of 12 tumours (83%).
- Moderate to diffuse immunostaining for cytokeratin 7 was identified in all 12 tumours (100%); on the other hand, only 3 of 12 (25%) tumours exhibited positive immunostaining for cytokeratin 20. Focal uroplakin III staining was found in 6 of 12 tumours (50%). In 5 cases (42%), focal to moderate CD10 immunoreactivity was observed. Immunohistochemical staining for OCT4 and CDX2 was completely negative in all tumours.
- All tumours exhibited chromosomal alterations similar to those commonly found in urothelial carcinoma in Urovysion fluorescence* in situ* hybridization (FISH) assays. Identical patterns of nonrandom X-chromosome inactivation in concurrent clear cell adenocarcinoma and urothelial neoplasia were observed in two informative female cases.

Sung et al. [6] stated the following.

- The appearance of clear cell adenocarcinoma of the urinary tract mimics the appearance of its counterpart in the female genital tract.
- Even though a number of theories had been promulgated about the origin of clear cell carcinoma of the urinary tract, its exact histogenesis had remained uncertain.

Sung et al. [6] concluded that their findings supported a urothelial origin for most clear cell adenocarcinomas of the urinary tract, despite their morphologic resemblance to certain Müllerian-derived tumours of the female genital tract.

Oliva et al. [2] reviewed the clinical, conventional pathological, and immunohistochemical characteristics of 13 neoplasms with exclusive, or predominant, morphologic features of clear cell carcinoma. They reported the following.

- On microscopic examination the most common pattern, present in all cases, was tubulocystic, with a papillary pattern, present in six tumours, and a predominant solid growth in one.
- Cells with abundant clear cytoplasm were conspicuous in nine tumours and hobnail cells were seen in eight.
- Four tumours exhibited focally recognizable patterns of transitional cell (urothelial) carcinoma in the available material. In five other tumours, pseudostratified epithelium reminiscent of transitional epithelium was present focally. Endometriosis was present in two cases. In two other cases benign cysts focally lined by ciliated epithelium and surrounded by elastosis were interpreted as most likely Müllerian.
- Immunohistochemistry was performed in 10 cases. All tumours stained for CA 125 (usually strong, ranging from focal to diffuse) and nine tumours stained for CK7 (usually strong and diffuse). CK20 was focally and weakly positive in four tumours and extensively positive in another.
- The same immunohistochemical panel was performed on 10 typical transitional cell carcinomas, 4 transitional cell carcinomas with gland differentiation, not otherwise specified, and 5 pure adenocarcinomas of the bladder (one of urachal origin). Minimal CA 125 positivity was seen in two transitional cell carcinomas. CA 125 staining was seen in the areas of gland differentiation in three of four transitional cell carcinomas and three of five pure adenocarcinomas but it was focal in most cases. All transitional cell carcinomas and transitional cell carcinomas with gland differentiation exhibited extensive CK7 positivity. In contrast, only one of four positive pure adenocarcinomas showed >5% CK7-positive cells. Although all groups showed CK20 positivity, the percentage of CK20 positive cells was higher in pure adenocarcinomas. Prostate specific antigen was negative in all tumours.
- The cytokeratin immunochemical staining profile of clear cell carcinomas of the bladder was closer to transitional cell carcinomas and transitional cell carcinomas with gland differentiation than to pure adenocarcinomas arguing against an unusual form of adenocarcinoma.

They made the following concluding statements.

- Their finding of CA 125 expression in bladder tumours of apparent urothelial origin contrasted with some studies that had regarded CA 125 expression as evidence for a Müllerian origin.
- The frequency of gland differentiation in transitional cell carcinomas and the rarity of vesical endometriosis could be taken to suggest that these tumours are mostly of urothelial derivation, but the strong female preponderance in their series argued for a Müllerian origin in at least some cases, and this was almost certain in the four cases with benign Müllerian components.
- In the absence of endometriosis or conventional foci of transitional cell carcinoma, it might be impossible to determine whether a tumour with the morphology of clear cell carcinoma is of Müllerian or transitional (urothelial) cell lineage, and at this time immunochemistry does not solve this problem.

**Figure 7:**
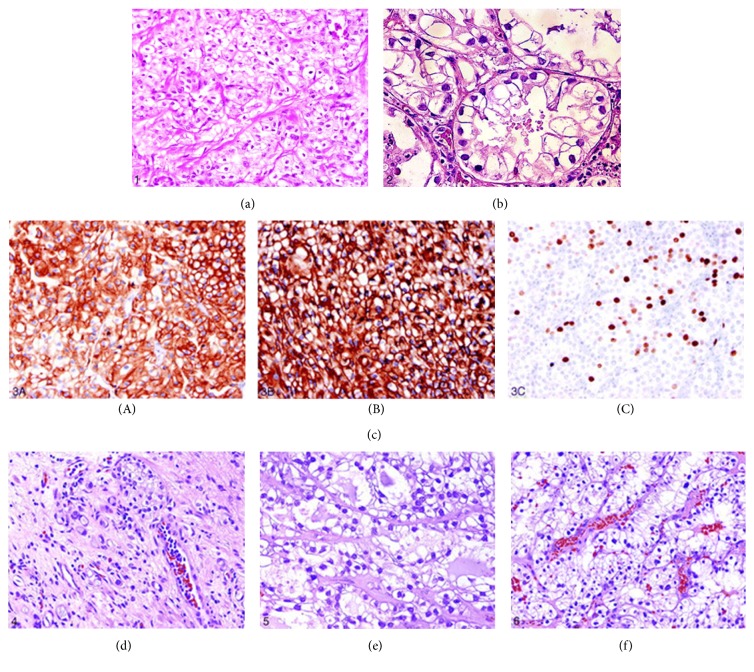
(a) Showing solid variant of clear cell adenocarcinoma of the bladder with clear cells rich in glycogen (hematoxylin-eosin original magnification ×200); (b) showing clear cell adenocarcinoma of the bladder with prominent tubulocystic pattern (hematoxylin-eosin magnification ×200); (c) immunohistochemical staining of clear cell adenocarcinoma cells for cytokeratin 7 (original magnification ×200) (A), epithelial membrane antigen (original magnification ×200) (B), and high MIB-1 activity (original magnification ×200) (C). Immunohistochemical stains for pan-cytokeratin and cytokeratin 20 were also positive (figures not shown); (d) showing nephrogenic adenoma small tubules and cysts lined by clear cells (hematoxylin-eosin, original magnification ×200); (e) showing clear cell adenocarcinoma of the ovary (for comparison) tubules and cysts lined by glycogen-rich clear cells (hematoxylin-eosin, original magnification ×200); (f) showing clear renal cell carcinoma confluent nests composed of cells with uniform clear cytoplasm (hematoxylin-eosin, original magnification ×200). Reproduced from Adeniran and Tamboli. [10]: with permission of American College of Pathologists.

Adeniran and Tamboli [10] in their review of clear cell carcinoma of the urinary bladder stated (see Figures 7(a), 7(b), 7(c), 7(d), 7(e), and 7(f) for various microscopic and immunohistochemical staining pictures of clear cell adenocarcinoma of bladder and some differential diagnoses) the following.

- These tumours are positive for pan-cytokeratin, cytokeratin 7, and CA 125 immunohistochemical stains.
- The natural history is poorly understood and patient outcomes remain unclear.
- At the time of their publication, surgery had remained the treatment of choice.

Sethi et al. [16] reported a 74-year-old woman who underwent transurethral resection of bladder tumour. Microscopic and immunohistochemical staining examinations were reported to be consistent with a diagnosis of clear cell adenocarcinoma of the urinary bladder. The patient was given intravesical mitomycin C and subsequently she underwent radical cystectomy. Examination revealed a microscopic focus of residual tumour in the bladder neck. However, all the margins were free of tumour. At six-month follow-up, the patient was reported to be doing well.

Sethi et al. [16] further stated the following.

- At the time of publication of their paper some authors had intimated that 41 cases of CCA had been reported [17].
- The histogenesis of CCA in the urinary bladder was still unclear and most information has been gained from single case reports and small case series as stated by Trabelsi and associates [18].
- PCCUBs were originally categorized as mesonephric adenocarcinoma by Konnak in 1973 [19].
- Later Young and Scully [5] in 1985 introduced the term CCA for these tumours, which has histological resemblance to the CCA of female genital tract of Müllerian origin.

Sung et al. [6] integrated molecular genetic evaluation by fluorescence* in situ* hybridization (FISH) and X-chromosome inactivation with conventional morphologic and immunohistochemical analyses in 12 patients, with clear cell adenocarcinomas in the urinary tract. They reported their results as follows.

- Concurrent urothelial carcinoma or urothelial carcinoma* in situ* was present in six cases (50%) and foci of cystitis glandularis were observed in four cases (33%).
- Neither intestinal metaplasia nor Müllerian component was identified in any case.
- Cytoplasmic expression of *α*-methylacyl-CoA racemase was demonstrable in 10 of 12 tumors (83%).
- Moderate to diffuse immunostaining for cytokeratin 7 was identified in all 12 tumours (100%), whereas only 3 of 12 (25%) tumours exhibited positive immunostaining for cytokeratin 20. Focal uroplakin III staining was seen in 6 of 12 tumors (50%). In five cases (42%), focal to moderate CD10 immunoreactivity was observed.
- Immunostains for OCT4 and CDX2 were completely negative in all tumours.
- In Urovysion fluorescence* in situ* hybridization assays, all the tumours displayed chromosomal alterations similar to those commonly found in urothelial carcinoma. Identical patterns of nonrandom X-chromosome inactivation in concurrent clear cell adenocarcinoma and urothelial neoplasia were identified in two informative female cases.

*They Concluded the Following*. Their findings supported a urothelial origin for most clear cell adenocarcinomas of the urinary tract, despite their morphological resemblance to certain Müllerian-derived tumors of the female genital tract.

Lu et al. [3] reported a 68-year-old female who underwent complete transurethral resection of the bladder tumour. The histological diagnosis was clear cell adenocarcinoma of the bladder. The muscle layer of the bladder was invaded by tumour cells (T2aN0M0). She refused to undergo partial or radical cystectomy or possible urinary diversion. She also refused to try chemotherapy or radiotherapy. She postoperatively had intravesical mitomycin C, 20 mg, once per week for 2 months. Initially her urinary symptoms were relieved. Nevertheless, three months after the surgery, a recurrent tumour was found at cystoscopy. She had a further CT scan which showed that bladder tumour had progressed and enlarged lymph nodes were found in right iliac and inguinal region. She insisted on having TUR-BTs rather than cystectomy.

After the second TUR-BT, the TNM stage was evaluated as T2bN1M0. The patient continued to have intravesical mitomycin C. She continued to have TUR-BT rather than cystectomy. The TNM stage had been advanced into T4aN2bM0. At a Follow-up 12 months after the last TUR-BT, she was still alive but with progression of the disease.

Lu et al. [3] reviewed relevant case reports published in the English literature to better evaluate the clinical characteristics of CCA. They stated that they had included a total of 38 case reports in their review [4, 5, 7, 11–15, 17, 20–25]. Lu et al. [3] summarized the results of the review as follows.

- The length of follow-up in these cases ranged from 8 months to 7 years.
- The mean age at presentation was 62.2 and the median age was 63 (range, 35–80).
- There were more female cases than male cases (21 females versus 16 males), which was different from transitional cell carcinoma (TCC).
- The usual presenting symptoms included visible haematuria, dysuria, recurrent urinary tract infection (UTI), and suprapubic pain.
- Patients rarely complained of local tumour effects or pain from local spread of the tumour [11].
- The most common locations from which the tumours originated were bladder neck and posterior wall, with incidence of 31.6% (12/38) and 26.3% (10/38), respectively. Other common locations included the trigone, lateral wall, and urethra.
- Dissimilar to urothelial carcinoma, most clear cell adenocarcinomas of the urinary bladder were large, solitary masses forming papillary or sessile structures [13].
- The histogenesis of CCA of the bladder remained controversial. In the older literature, the tumours in most cases were designated as “mesonephric adenocarcinoma,” but they lacked convincing evidence for a mesonephric origin [2].
- Some authors believe that CCA of urinary bladder arose from Müllerian elements in the bladder and were histogenetically identical to the female genital tract, because in some cases the neoplasms had been associated with vesical endometriosis or had arisen in Müllerian duct cysts or remnants in the bladder [4, 26]. This had also been considered an explanation for the observation that female incidence is more common. Nevertheless, a subsequent study presented evidence for urothelial origin in most clear cell adenocarcinomas of urinary tract, despite the morphologic resemblance to Müllerian-derived tumours of the female genital tract [6]. Furthermore, some authors [27, 28] stated that their studies suggested that CCA may be associated with nephrogenic adenoma because they share some similar histological features.
- The described microscopic features of CCA of the urinary bladder included admixtures of tubular glands, microcysts, papillae, and diffuse masses. The cells range from flat to hobnail and cuboidal with abundant, clear and glycogen-rich cytoplasm, often with significant nuclear atypia and mitotic activity [2, 7].
- Immunohistochemical staining of clear cell adenocarcinoma of the bladder was strongly positive for CK7 and variably positive for CK20, which is similar to typical urothelial carcinoma. CA-125 is typically positive, which is generally accepted as a marker of Müllerian differentiation [2, 6].
- The initial treatment for primary clear cell carcinoma of bladder was mainly surgical resection. All but 5 patients were firstly treated with a surgical resection (86.8%, 33/38). The surgical resection therapy entailed transurethral resection (12 patients, 31.6%), total cystectomy (11 patients, 28.9%), radical surgery (5 patients, 13.1%), partial cystectomy (3 patients, 7.9%), and other surgical resection (2 patients, 5.3%: one underwent diverticulectomy and one underwent unspecific tumour resection). Out of the 12 patients who had undergone TUR-BT for their initial surgical treatment, 5 patients did not receive other therapies. One of the 5 patients presented no evidence of disease in 4-year follow-up period. Other patients underwent additional radical surgery, total cystectomy, chemotherapy, and radiotherapy. Out of the 11 patients who had undergone total cystectomy for their initial surgical treatment, 7 patients did not receive other therapies. The longest follow-up period with no evidence of disease was 7 years. Four other patients received adjuvant chemotherapy and/or radiotherapy, which did not seem to have provided any additional effects because 2 of them died with disease in 2 years and 1 in 18 months. Four patients underwent radiotherapy as the initial treatment and two of them underwent total cystectomy subsequently. No advantageous effect of radiotherapy was observed based upon the fact that two patients died with disease in 8 months and 1 year, and the other two were alive with no evidence of disease in 18 months and 2 years [7, 11, 15]. Other authors treated some patients with chemotherapy (including cisplatin, doxorubicin, or cyclophosphamide) and/or radiotherapy (40–60 Gy total) as the adjuvant treatment but with uncertain effects [11, 13, 15]. With the addition of 2 patients who initially had radiotherapy, there were a total of 35 patients who underwent surgical resection. At the last follow-up, a total of 17 (48.6%) patients were alive with no evidence of disease and 8 (22.9%) patients were dead with disease, in 8 (22.9%) patients there were no data available, and 2 (5.7%) patients were alive with disease.
- Some authors [11, 13] had stated the following. (a) Generally, PCCUB is more malignant than common urothelial carcinoma, but more cases and longer follow-up periods are required to elucidate these points. (b) Lymph nodes and bone seem to be the most common metastatic sites for this disease [11, 13].

Lu et al. [3] concluded the following.

- In view of the fact that there are no characteristic symptoms for the clear cell adenocarcinoma of bladder, the diagnosis is mainly based on histopathology. Nevertheless, once the diagnosis is determined, the radical surgery should be recommended.
- Radiotherapy or chemotherapy may be helpful.
- Post-TUR intravesical therapy is of no help for preventing recurrence, although the TUR-BT had removed all visible tumours and even reached the muscular layer of the bladder.

Alvarez et al. [29] reported a 49-year-old woman who underwent transurethral resection of bladder tumour on two occasions. The final histological diagnosis of the tumour was clear cell adenocarcinoma with infiltration into the muscle layer of the bladder (pT2). It was associated with areas of high grade noninvasive transitional cell carcinoma. A CT scan showed retroperitoneal nodes and iliac adenopathy. She subsequently underwent further surgery with resection of an endometriotic ovarian cystic structure and an iliac adenopathy, which was identified during surgery as metastatic. Microscopic examination revealed that the adenopathy was entirely infiltrated by clear cell adenocarcinoma, without transitional component.

Postoperatively she received chemotherapy (cisplatin and gemcitabine). Radiotherapy was not considered because lymph nodes were present outside of the radiation field. During the fifth cycle, cisplatin was replaced with carboplatin due to a poorly controlled emetic syndrome. The best response after six cycles was radiological stabilisation, and the treatment was terminated. Radiological and clinical progression were observed three months later. Thus, a second line of treatment was commenced with docetaxel, and she received a total of five cycles, with radiological stabilisation after the third cycle and subsequent progression. Her treatment was suspended 27 months after the initial presentation (18 months after the cystoscopic diagnosis of the tumour), at which time it was decided to administer symptomatic and supportive treatment. Her tumour continued to progress and she developed multiple metastases including spinal metastasis for which she had radiotherapy. She subsequently died 38 months after her initial presentation (29 months after the cystoscopy finding of the tumour) as a result of tumour progression.

Alvarez et al. [29] stated the following.

- The clinical presentation of clear cell carcinoma of the urinary bladder is the same as that of urothelial tumours: voiding symptoms, acute urinary retention, and haematuria [23].
- The usual treatment of clear cell carcinoma of the urinary bladder is surgical, with partial or radical cystectomy [23]. The subsequent published follow-up had been short and rarely as long as 10 months with the longest follow-up at the time of publication of their paper being 30 months. In view of this the prognosis of clear cell carcinoma of the urinary bladder is difficult to estimate.
- However, a single case of locally advanced clear cell carcinoma of the urinary bladder which was treated by pelvic exenteration did not recur during 30 months of follow-up [30].
- In another case of clear cell carcinoma of the urinary bladder of extravesical spread, in which surgical treatment was not considered, the evolution was poor, and the patient died eight months pursuant to diagnosis as a result of metastatic disease [11].
- Only one case of radiotherapy treatment alone had been described, which resulted in complete response of the localized disease [31].
- Experience regarding chemotherapy as treatment for clear cell carcinoma of the urinary bladder is limited to an adjuvant setting. The drugs that had been used include cisplatin, 5-fluorouracil [23], carboplatin and methotrexate [32], and Adriamycin and cyclophosphamide [12] with or without radiotherapy. In view of the fact that the aforementioned instances are cases of adjuvant treatment which had little follow-up, they were unable to evaluate the disease sensitivity to chemotherapy.

Alvarez et al. [29] concluded the following.

- The combination of clear cell and transitional adenocarcinoma in this case did not allow for the role of each histology in determining the outcome and the poor response to therapy.
- In the case of the patient, the disease was not sensitive to chemotherapy. Though more than 24 months of survival after diagnosis of metastatic disease is longer than the outcome typically described in the literature, it could not be attributed to the chemotherapy treatment, because no objective response or clinical improvement was observed when the two lines of chemotherapy were administered. However, the patient improved on two occasions in response to the palliative radiotherapy treatment.
- From a pathological perspective, a combination of mixed histology and endometriosis might be of interest because each of these supports one of the two theories concerning the disease histogenesis. Unfortunately, the fact that the endometriosis was only in the ovary does not allow its connection with the neoplastic disease, and this combination could be a sporadic association.

Honda et al. [12] reported a case of mesonephric adenocarcinoma of the urinary bladder in a lady who had cystoscopy which revealed a nonpapillary tumour in her bladder. Histological examination of the bladder biopsy specimens revealed adenocarcinoma. She underwent total cystectomy and pelvic lymph node dissection. Honda et al. [12] reported that, histologically, the tumour was mainly composed of cells with eosinophilic cytoplasm and partly of cells with clear cytoplasm or hobnail-shaped cells, arranged in tubular or papillary structures, and infiltrated perivascular fat tissues. She died as a result of metastatic disease 22 months following her surgery.

Signori et al. [22] reported a case of clear cell adenocarcinoma of the urinary bladder in 54-year-old man. He underwent cystoscopy which revealed a tumour arising from the dome of his urinary bladder. Histological examination revealed the tumour to be chiefly composed of tubulocystic and papillary glands which were lined by glycogen-rich cubical or hobnail cells with clear to eosinophilic cytoplasm. The tumour infiltrated into the inner muscular layer (pT2 tumour). The patient was treated by partial cystectomy. The patient's eventual outcome was not reported.

Kurosaka et al. [23] reported a 52-year-old woman with a tumour which seemed to have arisen from the urinary bladder or the urethra, and bilateral iliac lymphadenopathy was seen. Cystoscopy examination revealed that the urethral mucosa was intact. Histopathological examination of the biopsy specimens revealed clear cell adenocarcinoma. She underwent radical cystourethrectomy with complete pelvic lymph node dissection and construction of a bilateral ureterocutaneoustomy. Histological examination of the tumour revealed adenocarcinoma which exhibited solid clear cells with glandular and papillary patterns. The tumour had infiltrated the perivesical structure (and hence was staged as pT3a), and metastases into multiple pelvic lymph nodes were identified (and hence staged as pN3). Postoperatively she received three courses of systemic combination chemotherapy with 5-fluorouracil (FU) and cisplatin along with a total of 45 Gy of radiotherapy during the second course of chemotherapy. There was no evidence of disease 28 months following the surgery.

Tazi et al. [30] reported a 19-year-old woman with a huge pelvic mass. She had transurethral biopsy of the tumour histological examination which showed the mesonephric nature of the mass. She underwent an anterior pelvic exenteration with total colpectomy, as well as radiotherapy. At her 30-month follow-up, she was alive without recurrence or metastasis.

Ito et al [14], in 1999, reported a case of clear cell adenocarcinoma of the urinary bladder in a 65-year-old man who had a walnut-sized nodular tumour in the anterior wall of the urinary bladder. He underwent radical cystoprostatectomy with urethral hemi-Koch pouch. Histological examination of the lesion revealed that the lesion was composed of poorly differentiated (G3) adenocarcinoma and clear cell carcinoma with diffuse sheet pattern of cells with abundant clear cytoplasm. Ito et al. [14] reported that the patient died of metastasis 18 months following his operation.

Doddamani et al. [32] reported a case of mesonephric or mesonephroid adenocarcinoma of the urinary bladder, which was treated by transurethral resection and subsequent chemotherapy but the outcome is not available to the author.

Doria et al. [33] reported the cytohistological findings of three cases of clear cell carcinoma (CCL) arising from the lower urinary tract. Filter and cytocentrifuge preparations of urine specimens of the 3 patients were studied and all the cases displayed numerous scattered aggregates or single tumour cells in an inflammatory background. The enlarged cells had abundant clear, wispy cytoplasm with discrete vacuolation. Hobnail and signet ring cells were seen. The nuclei exhibited granular/vesicular chromatin with prominent often multiple nucleoli. The tumours were reported to be histologically distinctive and typically had tubule-cystic configuration with variable proportions of papillary and diffuse patterns. One of the patients had died of metastatic carcinoma and two of the patients were at the time of publication of the paper alive and free of tumour. Doria et al. [33] stated that the cytohistological features of this carcinoma are characteristic and from their review they had concluded the lesion can be diagnosed by cytologic means. Out of the three cases reported by Doria et al. [33] two involved primary clear cell carcinoma of the urinary bladder. The first case reported by Doria et al. [33] was that of a 45-year-old woman who presented with a 5-month history of several episodes of visible haematuria. A diagnosis of clear cell carcinoma of bladder was made after urine cytology and partial resection of the tumour. She underwent radical cystectomy with pelvic exenteration. No metastases were found and 11 resected lymph nodes were negative. At the time of publication of the paper, the patient was alive without evidence of disease 15 months after her operation. The second case reported by Doria et al. [33] was a 63-year-old man who presented with haematuria. Based upon examination of his urine cytology specimen and transurethrally resected specimens of the tumour a diagnosis of clear cell carcinoma was made. He underwent partial cystectomy and intravesical Bacille Calmette Guerin (BCG) but the tumour recurred 5 years later. He underwent radical cystectomy. He received adjuvant chemotherapy and radiotherapy; however, the tumour persisted and the patient died of metastatic disease 7 years pursuant to his presentation.

Zachos et al. [34] in 2006 reported a case of primary small cell bladder carcinoma in Tumori; however, details of the case report are not available to the author. They stated that, recently, a two-stage system for limited and extensive small cell bladder carcinoma has been suggested in analogy to the practiced staging and treatment of small cell lung carcinoma.

In summary, with regard to the sex of the 46 patients who were reported to have had PCCUB, 26 were female and 17 male but the sex of the remaining three patients was not available to the author. With regard to outcome of the 46 patients reported with PCCUB following treatment, details of outcome were not available to the author with regard to 10 patients. However, of the remaining 36 patients, 14 patients had died of their disease at intervals which ranged between 6 months and 7 years following treatment; 18 were alive without disease at intervals which ranged between 9 months and 5 years; 2 were alive with disease (one of them at 63-month follow-up) (see Table 1 for details). The aforementioned outcomes are a general outcome which does not take into account the histological grade and clinical stage of the tumours and would not explain the biological behaviour of PCCUB with regard to histological grade, clinical stage, and type of treatment. There is therefore a need to further study the biological behaviour of PCCUB in a well-planned multicentric study of PCCUB.

## 4. Conclusions

PCCUBs are rare. The diagnosis of PCCUB is based upon characteristic histological and immunohistochemical features. A thorough radiological assessment is required to exclude a primary tumour elsewhere. There is no consensus opinion regarding the treatment of this tumour. Urologists and oncologists should report all cases of PCCUBs they encounter and enter them into a multicentric trial to establish the best treatment option.

## Figures and Tables

**Figure 1:**
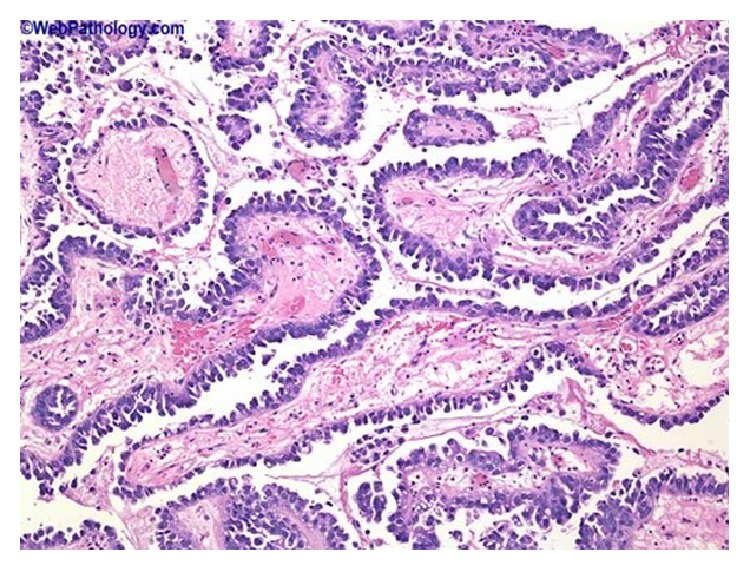
This shows clear cell adenocarcinoma of the urinary bladder, papillary tumour with prominent hobnailing. Reproduced from PathologyOutlines.com [1] which obtained it from webpathology.com with permission from webpathology.com and from PathologyOutlines.com.

**Figure 2:**
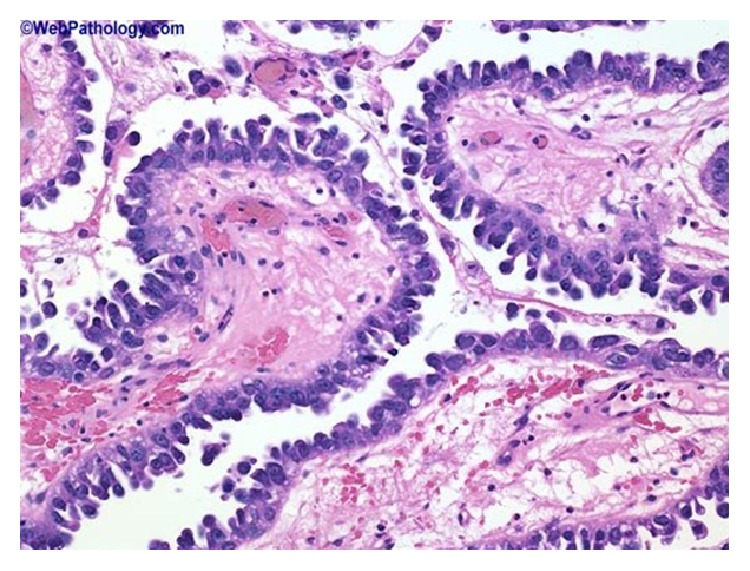
This shows clear cell adenocarcinoma of the bladder illustrating a papillary tumour with prominent hobnailing; the tumour cells are arranged in tubular structures, papillae, cysts, or diffuse sheets. This example shows a predominance of dilated papillary structures lined by hobnail cells with scant cytoplasm. Cytologic atypia is not striking. Reproduced from PathologyOutlines.com [1] which obtained it from webpathology.com with permission from PathologyOutlines.com and webpathology.com.

**Figure 3:**
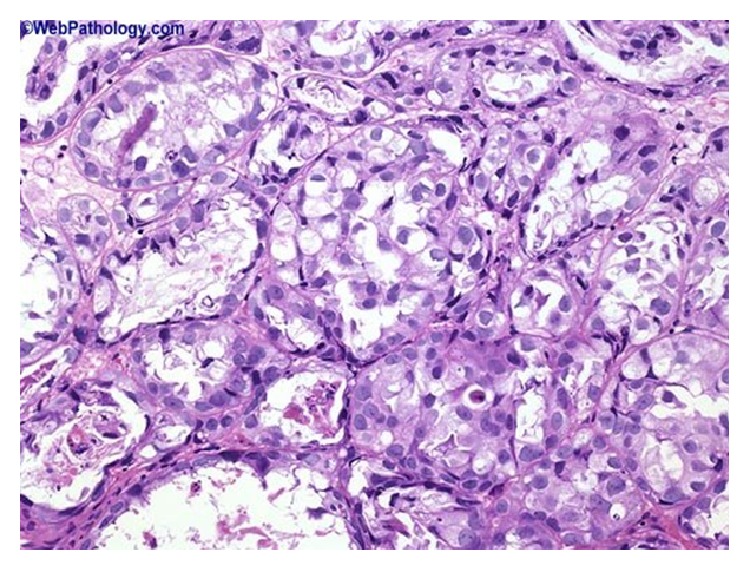
This shows clear cell carcinoma of the urinary bladder which contains tubules and nests of cells with clear cytoplasm: in this example of clear cell adenocarcinoma, the tumour cells are arranged in tubules and solid nests and have abundant clear cytoplasm. Reproduced from PathologyOutlines.com [1] which obtained it from webpathology.com with permission obtained from both PathologyOutlines.com and webpathology.com.

**Figure 4:**
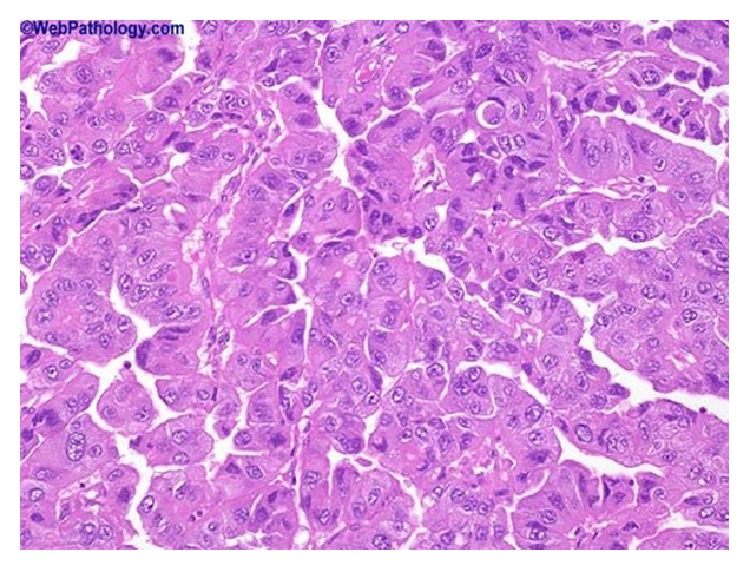
This shows clear cell adenocarcinoma of the urinary bladder which contains prominent papillary component with focal eosinophilic cytoplasm. Some cases have a prominent papillary component as seen here. Reproduced from PathologyOutlines.com which obtained it from webpathology.com with permission obtained from both PathologyOutlines.com and webpathology.com.

**Figure 5:**
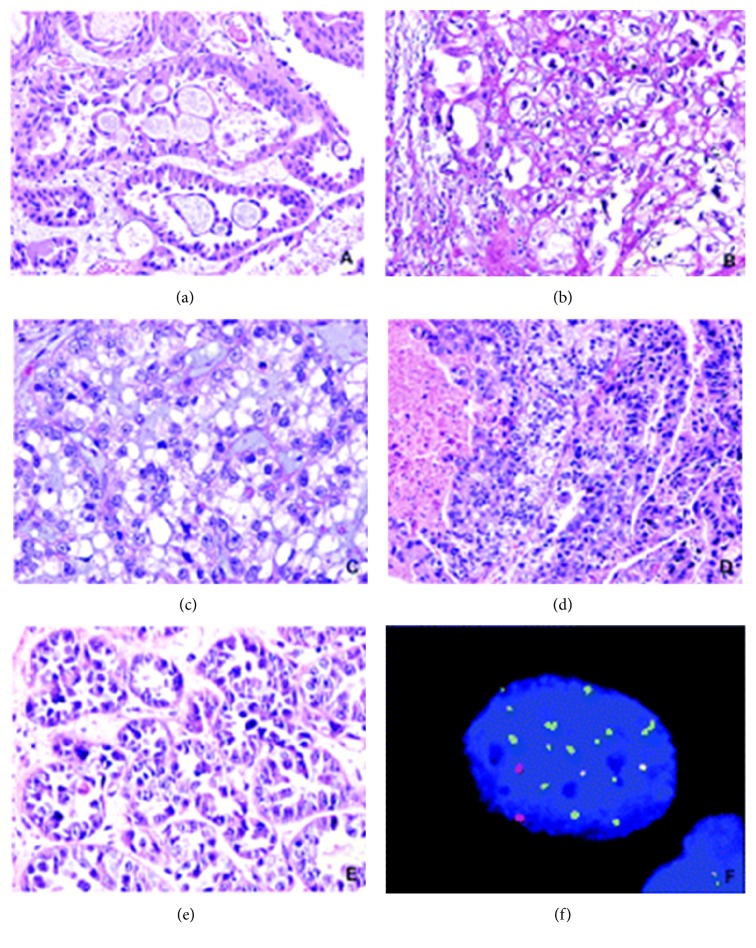
This figure shows various types of images on clear cell adenocarcinoma of the urinary tract. (a) The tumour showed tubulocystic structure with characteristic lining hobnail cells. (b) Polygonal tumour cells revealed abundant clear cytoplasm. (c) Solid diffuse growth of clear cell adenocarcinoma, exhibiting marked cellular pleomorphism, nucleolar prominence, and mitotic activity. (d) Tumour necrosis was observed within the cystic space. The solid and tubular tumour components showed clear or eosinophilic cytoplasm. (e) Typical tubular formation with inner lining pleomorphic neoplastic cells. (f) A typical tumour cell from clear cell adenocarcinoma of bladder showed chromosomal abnormalities detected by FISH. The cell showed gaining of chromosomes 7 (green) and 17 (aqua) but with normal copy of numbers of chromosomes (red) and 9p21 (gold). Reproduced from PathologyOutlines.com which obtained it from Sung et al. [6] with permission from PathologyOutlines.com and American Association for Cancer Research and Clinical Cancer Research.

**Figure 6:**
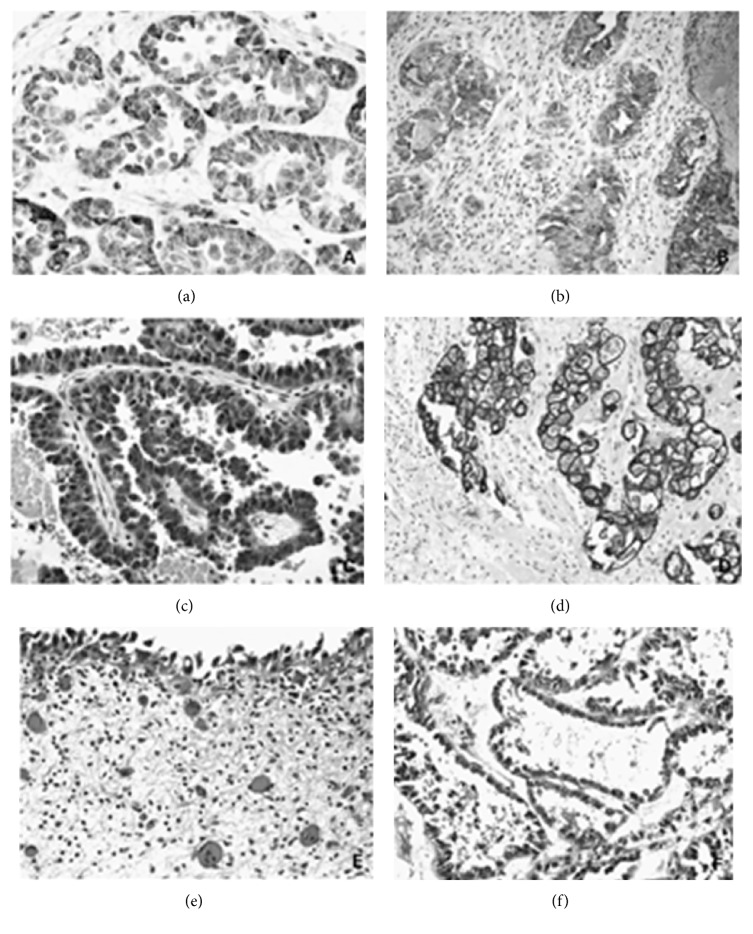
This figure shows various images of immunohistochemical staining and microscopic appearance of clear cell adenocarcinoma of the urinary tract and concurrent urothelial carcinoma in situ; (a) tumour cells were positively stained by AMACR, representing cytoplasmic staining in the tubular components. (b) Diffuse membranous and cytoplasmic expression of CD10; (c) positive uroplakin III expression in clear cell adenocarcinoma; (d) CK7 was strongly positive. ((e) and (f)) A female patient represented with concurrent urothelial carcinoma* in situ* (e) and clear cell adenocarcinoma. (f) Reproduced from PathologyOutlines.com [1] which obtained it from Sung et al. [6] with permission from PathologyOutlines.com and Clinical Cancer Research.

**Table 1:** List of some reported cases of primary clear cell carcinoma of the urinary bladder and outcome.

Case number	Reference	Age	Sex	Site	Treatment	Outcome
1	Dow and Young [15]	43	Male	Right lateral wall, neck	Radiotherapy and total cystectomy	Died with disease, 1 year

2	Matsuoka et al. [11]	68	Female	Neck	Transurethral resection and radiotherapy	Outcome not available to author

3	Drew et al. [7]	54	Female	Neck	Total cystectomy	No evidence of disease after 2 years

4	Drew et al. [7]	71	Female	Neck/urethra	Radiotherapy	No evidence of disease after 2 years

5	Drew et al. [7]	53 years	Female	Right ureteric orifice	Total cystectomy	Died with disease, 9 m

6	Matsuoka et al. [11]	68 years	Female	Posterior, left lateral wall	Partial cystectomy	No evidence of disease after 3 years

7	Matsuoka et al. [11]	Not available to author	Not available to author	Not available	Total cystectomy	Died with disease, 1 year

8	Drew et al. [7]	70 years	Female	Neck	Radical surgery	No evidence of disease, 10 months

9	Matsuoka et al. [11]	63 years	Male	Left lateral wall, trigone	Total cystectomy	No evidence of disease, 7 years

10	Matsuoka et al. [11]	57 years	Female	Neck	Radiotherapy and total cystectomy	No evidence of disease, 18 months

11	Young and Scully [5]	78 years	Female	Trigone	Transurethral resection	No evidence of disease, 4 years

12	Matsuoka et al. [11]	61 years	Female	Posterior wall	Partial cystectomy	No evidence of disease, 5 years

13	Matsuoka et al. [11]	62 years	Female	Left ureteric orifice	Transurethral resection	Not available

14	Matsuoka et al. [11]	62 years	Female	Posterior wall, trigone	Total cystectomy	No evidence of disease, 2 years

15	Matsuoka et al. [11]	73 years	Female	Posterior, anterior wall, neck, urethra	Radical surgery	Not available

16	Matsuoka et al. [11]	53 years	Male	Posterior, anterior wall	Transurethral resection, radiotherapy	No evidence of disease

17	Matsuoka et al. [11]	78 years	Female	Right lateral wall, neck.	Transurethral resection, chemotherapy, total cystectomy	Not available

18	Matsuoka et al. [11]	72 years	Male	Trigone, posterior, left lateral wall.	Total cystectomy	Died with disease, 2 years

19	Chor et al. [4]	35 years	Female	Posterior wall	Total cystectomy	Not available

20	Matsuoka et al. [11]	67 years	Male	Trigone, posterior wall.	Total cystectomy	No evidence of disease, 14 months

21	Drew et al. [7]	63 years	Male	Not available	Chemotherapy, radiotherapy	Died with disease, 18 months

22	Drew et al. [7]	78 years	Female	Not available	Radical surgery	No evidence of disease, 12 months

23	Drew et al. [7]	50 years	Male	Not available	Transurethral resection, radical surgery	Alive with persistence/progression of disease, 63 months

24	Drew et al. [7]	43 years	Male	Not available	Transurethral resection	No evidence of disease, 30 months

25	Matsuoka et al. [11]	80 years	Male	Neck, left ureteric orifice.	Transurethral resection, chemotherapy	No evidence of disease, 25 months

26	Ito et al. [14]	65 years	Male	Anterior wall	Total cystectomy, chemotherapy	Died with disease, 18 months

27	Mai et al. [20]	68 years	Female	Trigone	Radical surgery	Not available

28	Honda et al. [12]	59 years	Female	Trigone, neck	Total cystectomy, chemotherapy	Died with disease, 2 years

29	Matsuoka et al. [11]	59 years	Male	Posterior wall	Radiotherapy	Died with disease, 8 months

30	Moinzadeh et al. [21]	69 years	Male	Diverticulum, right ureteric orifice	Diverticulectomy	No evidence of disease, 1 year

31	Signori et al. [22]	54 years	Male	Dome of bladder	Partial cystectomy	Died with disease, 6 months

32	Kosem and Sengul [13]	55 years	Male	Left lateral wall	Transurethral resection and chemotherapy	Died with disease, 18 months

33	Kurosaka et al. [23]	52 years	Female	Neck	Total cystectomy, radiotherapy, chemotherapy	No evidence of disease, 28 months

34	Lum [24]	68 years	Male	Not available	Tumour resection	Not available

35	Adams et al. [25]	62 years	Female	Neck, trigone, urethra	Transurethral resection, radical surgery	No evidence of disease, 9 months

36	Sun et al. [17]	56 years	Female	Not available	Radical surgery	No evidence of disease, 9 months

37	Drew et al. [7]	69 years	Male	Not available	Transurethral resection	Not available

38	Lu et al. [3]	68 years	Female	Posterior wall, neck, urethra	Transurethral resection	Alive with persistent/progressive disease.

39	Sethi et al. [16]	74 years	Female	Bladder neck, anterior and posterior walls	Transurethral resection, intravesical mitomycin c, and radical cystectomy	Alive 6 months without evidence of disease

40	Alvarez et al. [29]	49 years	Female	Neck and left half of trigone and metastases to iliac nodes and endometriosis like ovarian cystic structure	Transurethral resection, surgical resection of endometriosis ovarian cystic structure and iliac nodes plus chemotherapy cisplatin and gemcitabine 5 cycles plus 6th cycle with carboplatin and gemcitabine and then 2nd line chemotherapy docetaxel 5 cycles and palliative radiotherapy	Died with disease 2 years after treatment was started

41	Honda et al. [12]	59 [12]	Female	Trigone and bladder neck and deep into muscle layer	Total cystectomy and pelvic lymph node dissection	Died of metastatic disease 22 months after surgery

42	Pegoraro et al. [31]	Details not available	Details not available	Details not available	Details not available	Details not available

43	Doddamani et al. [32]	Details not available	Details not available	Details not available	Transurethral resection of tumour and subsequent chemotherapy	Details not available (9th reported case)

44	Tazi et al. [30]	19 years	Female	Pelvis/bladder	Anterior exenteration, colpectomy, and radiotherapy	Alive and well with no tumour after 30 months

45	Doria et al. [33]	45 years	Female	Bladder base/trigone	Radical cystectomy with pelvic exenteration	Alive without disease 15 months after surgery

46	Doria et al. [33]	63 years	Male	Right wall and anterosuperior wall of the bladder	Transurethral resection; partial cystectomy and intravesical BCG and later radical cystectomy, adjuvant chemotherapy and radiotherapy	He died of metastatic disease 7 years after presentation

47	Zachos et al. [34]	Details not available	Details not available	Details not available	Details not available	Details not available
